# Internet-Based Cognitive Behavior Therapy Only for the Young? A Secondary Analysis of a Randomized Controlled Trial of Depression Treatment

**DOI:** 10.3389/fpsyt.2020.00735

**Published:** 2020-07-24

**Authors:** Alexander Pabst, Margrit Löbner, Janine Stein, Melanie Luppa, Anette Kersting, Hans-Helmut König, Steffi G. Riedel-Heller

**Affiliations:** ^1^ Medical Faculty, Institute of Social Medicine, Occupational Health and Public Health (ISAP), University of Leipzig, Leipzig, Germany; ^2^ Department of Psychosomatic Medicine and Psychotherapy, University of Leipzig, Leipzig, Germany; ^3^ Department of Health Economics and Health Services Research, University Medical Centre Hamburg-Eppendorf, Hamburg, Germany

**Keywords:** late-life depression, internet-based cognitive behavior therapy, age, randomized controlled trial, primary care, e-mental health

## Abstract

**Background:**

Late-life depression is a major public health concern, driving the development of complementary treatment options. This study investigates the effectiveness and acceptability of internet-based Cognitive Behavioral Therapy (iCBT) in older individuals (60+ years) compared to younger age groups.

**Materials and Methods:**

Secondary analysis of a cluster-randomized controlled trial with 647 (18–82 years; mean 43.9) mild to moderately severe depressed primary care patients receiving either iCBT + treatment as usual (TAU) or TAU alone. Severity of depression was measured by the Beck Depression Inventory (BDI-II) at baseline, 6 weeks and 6 months. Intention-to-treat analysis in three age groups (18–39 years, n = 264; 40–59 years, n = 300; 60+ years, n = 83) was performed, using mixed-effects regression models to quantify treatment effect.

**Results:**

No age differences in the effectiveness of iCBT were found. Patients in the intervention group consistently showed a greater reduction in depression severity than controls in all three age groups and at both follow-ups. Effect sizes ranged from *d* = 0.30 (40–59 years, 6 weeks) to *d* = 1.91 (60+ years, 6 months). Uptake of the intervention was banded around 70% with no differences between age groups (*χ²* = 0.18, *p* = .915). The mean number of completed modules increased with age (*χ²* = 18.99, *p* = .040).

**Discussion:**

iCBT is equally effective in both younger and older individuals, thus providing a valuable complementary element of routine late-life depression care.

**Clinical Trial Registration:**

DRKS-ID: DRKS00005075 https://www.drks.de/drks_web/navigate.do?navigationId=trial.HTML&TRIAL_ID=DRKS00005075

## Introduction

Depressive disorders are highly prevalent among the general population and a major contributor to poor health and global disability. According to estimates of the World Health Organization (WHO), over 300 million people worldwide suffer from depression, equivalent to 4.4% of the world’s population (1).

Depression is also a common condition in the elderly (2). Recent findings from the Survey on Health, Ageing and Retirement in Europe showed a prevalence rate of 29% for depression in a population-based sample of individuals aged 65 years and older (3). Among the oldest-old (75+ years), a meta-analysis found pooled prevalence rates of 7.2% for major depression and 17.1% for depressive disorders (4). Late-life depression is not only associated with reduced quality of life for the individual, but also with increased morbidity and mortality (5–7). For instance, there is evidence for a high comorbidity between depression and the development of cognitive impairment and dementia in later life (8–12), which emphasizes the need for appropriately targeted interventions in older people. Even though the prevalence is high, and the individual and global health burden is substantial, there still is a huge gap in the use of mental health services for late-life depression in Europe (3).

The use of Internet-based interventions as a complementary therapy or self-help option for mental health disorders has been the subject of numerous studies in recent years. Their development is predominantly based on cognitive behavioral therapy and therefore often referred to as iCBT. Meta-analyses regarding guided and unguided iCBT in mixed-age samples show substantial evidence for its effectiveness in improving symptoms of many mental disorders (13, 14), in particular depression (15–17).

Younger patients generally more often express a strong preference for Internet-based interventions than do older patients (18). However, although Internet use and health-related help-seeking behavior has increased considerably in old age in recent years (19–21), studies on the acceptability and effectiveness of iCBT for depression in older people are scarce. A recent systematic literature review on the use of technology in mental health care for depressed elderly people included nine studies, with findings mostly referring to telehealth interventions such as telephone or Skype (22). Only one study referred to an Internet-based intervention (23). The authors reported a positive effect in reducing depressive symptoms in patients with type 1 or type 2 diabetes compared to a waiting list control. However, the sample predominantly included mid-age adults (mean age: 50 years, SD 12) and effectiveness was not analyzed by age. Another recently published meta-analysis by Xiang et al. (24) directly focused on iCBT for treating depressive symptoms in late-life. The authors identified nine studies, including a total of 1,272 participants with an average age of 66 years. The mean between-group effect size for iCBT compared to control was large (Cohen’s *d* = 1.18) and the majority of included studies were reported to show clinically significant improvements in favor of iCBT. However, the authors emphasize the preliminary character of their findings due to the limited number of studies, low overall study quality, and a lack of controls with inclusion of non-clinical samples in some studies. In addition, seven out of the nine studies were conducted in Australia, and it remains unclear whether the findings generalize to other countries.

Even though these first findings were promising, Xiang et al. (24) call out for more studies with rigorous trial design to substantiate earlier results. The present study aims to add to the existing literature by performing a secondary analysis of a randomized controlled trial (RCT) on the effectiveness of a self-guided iCBT program as an adjunct element of depression treatment in primary care. The original study found that iCBT effectively reduces depressive symptoms in mild to moderately severe depressed patients in the short and long-term (25). Results also pointed to a long-term improvement in self-efficacy and quality of life. Here, we aim to disaggregate the findings by age in order to evaluate the following research questions: (1) How effective is iCBT as add-on self-help in elderly primary care patients (60+ years) with mild to moderately severe depression compared to younger age groups?, and (2) How accepted is iCBT in elderly patients compared to younger age groups?

## Materials and Methods

This study is a secondary analysis of the cluster-randomized @ktiv trial; a detailed description of the design, trial procedures, flow of participants, and main results have been reported previously (25). The trial was approved by the IRBs (Ethics Committees) of the University of Leipzig (reference number 222/14ff) and of the Australian National University (reference number 2013/342). In addition, the data handling protocol for the trial was approved by the Commissioner for Data Privacy of the University of Leipzig (date: 18.12.2013).

### Trial Procedures

In short, a total of 647 mild to moderately severe depressive patients aged 18 years and older were recruited from 112 general practices in three German federal states, of which 320 have been randomized to the intervention group (IG) and 327 to the control group (CG). Depression status for inclusion was based on ICD-10 diagnosis and a positive screening on the Patient Health Questionnaire [PHQ-9; (26)]. Patients with severe depressive disorder, bipolar or psychotic disorders, and drug abuse were excluded, as were those who already received psychotherapy at the time of recruitment. Data were collected between February 2014 and August 2015 and included a baseline assessment and two follow-ups at six weeks and at six months, respectively.

### Interventions

In the intervention condition, participants received usual primary care combined with self-guided iCBT (TAU+iCBT) provided by the German version of the Internet-based, self-management program *moodgym* (27). *Moodgym* has been shown to effectively reduce symptoms of depression in the primary care setting (25, 28). The program consists of five interactive modules to be completed sequentially, each focusing on a specific aspect of iCBT including feelings, thoughts, changing dysfunctional thinking, destressing, and relationships (29). The German version was developed by specialists in mental health care at ISAP, taking German cultural norms in the translation process into account. *Moodgym* was exclusively available for participants during the trial and became available free-of-charge afterwards. Participants received detailed information on the study and instructions for accessing *moodgym* online *via* a personal login from their GP during the recruitment visit. GPs supported the use of self-management iCBT during the study by recommending it as an adjunct element of treatment.

In the control condition, participants were treated as usual (TAU) by their GP. There were no constraints on TAU, which allowed for specialized in- and out-patient care during follow-up if required.

### Measures

Clinical data were collected through self-report questionnaires, administered to participants during the recruitment visit at baseline and mailed to them 6 weeks and 6 months after baseline.

#### Depression

The primary outcome for this substudy was severity of depression as measured by the German-language version of the Beck Depression Inventory (30).

#### Covariates

As confounding variables, we assessed baseline sociodemographic information on gender, age, marital status, domicile, education, vocational qualification, and employment status. In addition, we included items on the history of psychological problems and depression, and on the existence of symptoms of a comorbid panic disorder (PD) and/or a generalized anxiety disorder (GAD) according to ICD-10 (31). Finally, we included a dummy variable for self-reported medication intake due to psychological distress at 6 weeks and/or 6 months in order to control for possible differences in the medication profile in the context of TAU during follow-up.

#### Uptake and Adherence

From pseudonymised computer log files, we obtained data on iCBT uptake (yes vs. no), the number of completed iCBT modules (ranging from 0 to 5), and the overall usage time (up to six weeks vs. longer than six weeks).

### Statistical Analysis

We performed intention-to-treat analysis, using multiple imputation by chained equations to account for missing baseline data, creating 25 completed datasets that were used for all analysis (32). The association between iCBT treatment and depression severity was assessed using multilevel mixed-effects linear regression models. These models included the baseline BDI-II score as a covariate, with treatment, time, treatment-by-time interaction and all other covariates as fixed effects, and comprised a random intercept to account for heterogeneity within patients over time. Analyses were stratified by age (18–39 years, 40–59 years, and 60+ years) to determine differences in the effect of iCBT treatment on depression severity over the life course. This stratification was chosen to be comparable to other literature with regard to iCBT in older adults [e.g., (33–35)]. In addition, the overall effect modification by age was evaluated by testing a 3-way interaction between treatment, time, and continuous and categorical age in the unstratified model. Finally, age differences in iCBT uptake and adherence were tested using Pearson’s *χ²* test (qualitative) or Cuzick’s (36) nonparametric test for trends across ordered groups (quantitative).

Descriptive statistics are presented as frequencies with percentages or means with corresponding standard deviations. Treatment effect was expressed as adjusted mean group differences and 95% confidence intervals in BDI-II score at 6 weeks and 6 months follow-up. In addition, we calculated effect sizes (Cohen’s *d*) for mean comparisons, using the adjusted mean scores obtained from the mixed models. We also estimated rates for remission (BDI-II score ≤ 9) and response (> 50% reduction in BDI-II score compared to baseline) for both groups at six months (37).

All analyses were performed using the Stata 15.1 MP software package (StataCorp LP, College Station, TX). Standard errors in all models were calculated using a clustered sandwich estimator with GP code as clustering variable.

## Results

### Baseline Characteristics

Of the 647 participants enrolled in the trial, 264 were in the 18–39 years age-group, 300 were in the 40–59 years age-group, and 83 were in the 60 years or older age-group. Total age ranged from 18 to 82 years, mean 43.9 years (± 13.7).

Baseline characteristics are shown by age and treatment condition in **Table 1**. Unlike the other age groups, IG participants in the 60+ year-old group were more frequently female and slightly younger than CG participants. The oldest age group reported less frequently to live with others (e.g., children, relatives) than did the younger age groups. Participants aged 40 to 59 years had fewer years of schooling than younger and older participants, and participants aged 60 years and older more often reported a university degree than others. IG participants in all age groups reported less frequently to have ever been depressed and more frequently to have current symptoms of an anxiety disorder.

**Table 1:** Baseline characteristics by age and treatment group (*n* = 647).

	18–39 years (*n* = 264)		40–59 years (*n* = 300)		60–82 years (*n* = 83)	
	CG	IG	CG	IG	CG	IG
	(*n* = 98)	(*n* = 166)	(*n* = 167)	(*n* = 133)	(*n* = 62)	(*n* = 21)
**Sociodemographic**						
Female	64 (65.3)	107 (64.5)	116 (69.5)	96 (72.2)	42 (67.7)	18 (85.7)
Age, mean ± SD	30.3 ± 5.3	30.1 ± 5.6	50.3 ± 5.6	49.2 ± 5.7	67.1 ± 6.4	63.2 ± 4.0
Marital status						
married	20 (20.4)	37 (22.3)	89 (53.3)	77 (58.1)	39 (62.9)	13 (61.9)
unmarried	75 (76.5)	122 (73.5)	39 (23.3)	23 (17.0)	5 (8.1)	3 (14.3)
divorced/widowed	3 (3.1)	7 (4.2)	39 (23.4)	33 (24.9)	18 (29.0)	5 (23.8)
Domicile^a^						
alone	24 (24.5)	43 (25.7)	42 (25.1)	22 (16.2)	18 (29.0)	7 (33.3)
with partner/spouse	54 (55.1)	78 (47.1)	107 (64.1)	100 (75.5)	40 (64.5)	13 (61.9)
with others	50 (51.0)	84 (50.8)	51 (30.5)	66 (49.9)	4 (6.5)	4 (19.0)
**Educational**						
Education						
≤ 10 years	66 (67.3)	90 (54.0)	133 (79.6)	102 (76.5)	38 (61.3)	12 (57.1)
> 10 years	32 (32.7)	76 (46.0)	34 (20.4)	31 (23.5)	24 (38.7)	9 (42.9)
Vocational qualification						
none/vocational training	79 (80.6)	120 (72.2)	112 (67.1)	85 (63.6)	33 (53.2)	9 (41.5)
secondary vocational education	10 (10.2)	21 (12.7)	34 (20.5)	24 (18.3)	6 (9.7)	5 (25.0)
university degree	9 (9.2)	25 (15.1)	21 (12.4)	24 (18.1)	23 (37.1)	7 (33.5)
Employment						
full-time	42 (42.9)	73 (44.0)	72 (43.1)	77 (58.1)	3 (4.8)	3 (14.2)
part-time/marginal	21 (21.4)	30 (18.0)	38 (22.8)	25 (19.0)	3 (4.8)	1 (4.8)
unemployed/retired/other	35 (35.7)	63 (38.0)	57 (34.1)	31 (22.9)	56 (90.4)	17 (81.0)
**Clinical**						
Ever psychological problems	63 (64.7)	96 (58.0)	114 (68.1)	100 (74.9)	48 (77.4)	16 (74.3)
Ever depression	75 (76.5)	96 (57.6)	135 (80.8)	92 (68.8)	48 (78.2)	14 (66.7)
Symptoms of GAD	23 (23.7)	50 (30.4)	29 (17.3)	40 (30.2)	4 (5.7)	5 (24.2)
Symptoms of PD	20 (20.5)	49 (29.3)	48 (28.7)	44 (33.1)	11 (17.4)	3 (12.8)
Symptoms of GAD and/or PD	36 (36.3)	83 (49.8)	59 (35.6)	67 (50.0)	13 (20.6)	7 (33.5)

Entries are n (%) unless indicated differently. CG, Control group (TAU); IG, Intervention group (iCBT+TAU); GAD, Generalized anxiety disorder according to ICD-10; PD, Panic disorder according to ICD-10. ^a^Multiple responses possible.

In addition, self-reported data on TAU during follow-up showed that 42% of IG (69/166) and 49% of CG participants (48/98) in the age-group of 18 to 39 years received medication due to psychological distress at 6 weeks and/or at 6 months. In the middle age group, 56% of the IG (74/133) and 70% of the CG (117/167) did so. In the 60+ years age group, 76% of IG patients (16/21) and 68% of CG patients (42/62) reported taking medication at 6 weeks and/or 6 months follow-up.

### BDI-II Scores

In the youngest and oldest age groups, IG participants had higher baseline BDI-II scores compared to CG participants (**Table 2**). Over time, BDI-II scores continuously decreased in all age groups, with consistently larger decreases in the IG as compared to the CG. One exception is seen among CG participants aged 60 years and older, where the mean BDI-II score slightly increased from baseline to 6 weeks follow-up.

**Table 2:** Average BDI-II scores over time by age and treatment group.

	**18–39 years**		**40–59 years**		**60–82 years**	
	*n*	mean (SD)	*n*	mean (SD)	*n*	mean (SD)
**CG**						
Baseline	98	21.6 ± 10.0	167	21.2 ± 10.5	62	16.1 ± 8.2
6 weeks	82	19.0 ± 10.3	157	20.1 ± 11.4	57	16.5 ± 9.9
6 months	69	14.0 ± 9.9	146	19.3 ± 12.1	54	14.8 ± 10.6
**IG**						
Baseline	166	25.2 ± 9.0	133	22.2 ± 8.4	21	19.4 ± 9.5
6 weeks	125	19.1 ± 10.5	105	18.0 ± 9.6	16	12.9 ± 8.6
6 months	96	14.4 ± 11.4	96	14.7 ± 11.0	18	10.8 ± 8.5

CG, Control group (TAU); IG, Intervention group (iCBT+TAU).

### Treatment Effect Across Age Groups

**Figure 1:**
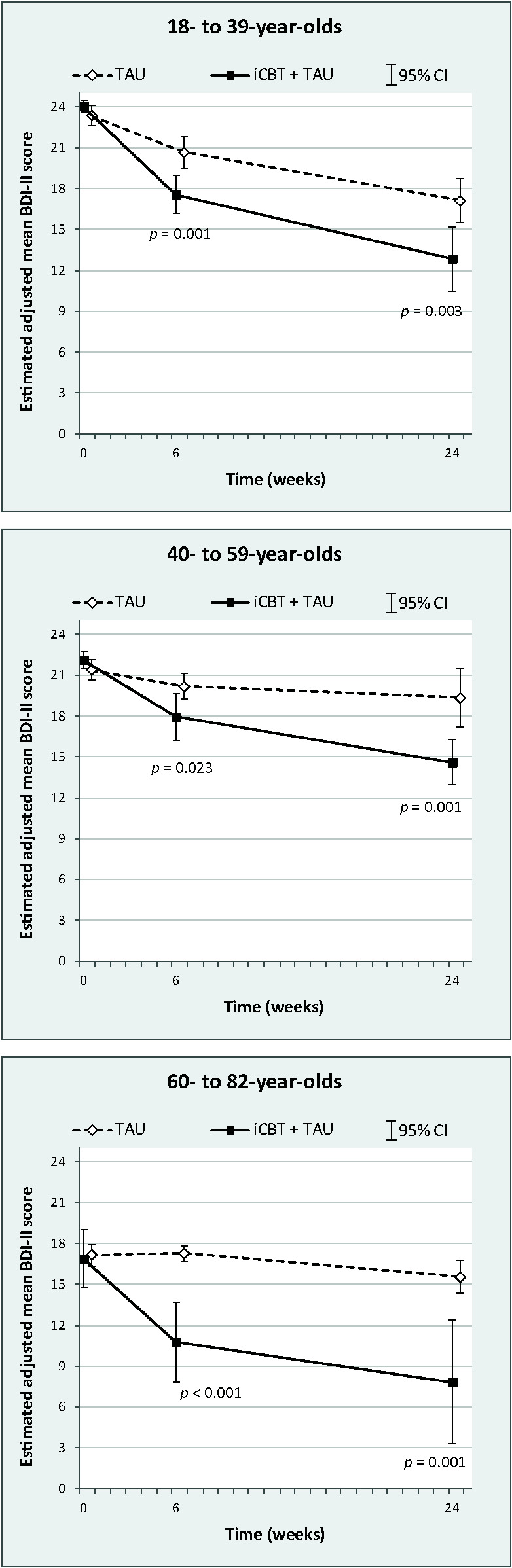
Estimated adjusted mean BDI-II scores with corresponding 95% confidence intervals by study group and time, and stratified by age. P-values for comparison of scores between groups at follow-up.

Results of the stratified mixed models revealed significant group differences in the mean BDI-II scores at both follow-ups in all three age groups (**Table 3**). After adjustment for baseline BDI-II score and covariates, IG participants consistently experienced a significantly greater reduction in depression severity than controls. The estimated mean group differences in favor of iCBT ranged from -2.26 points (95% CI: -4.22 to -0.31) in the 40-59 years age-group at six weeks to -7.73 points (95% CI: -12.42 to -3.05) in the 60 years or older age-group at six months (**Figure 1**). The corresponding effect sizes were still small (*d* < 0.5) in those younger than 60 years but large (*d* > 1.0) in older participants. Overall, age did not modify the treatment effect, as suggested by the nonsignificant 3-way interaction with continuous (*p*_interaction group X time X age_ = .372) and categorical (*p*_interaction group X time X age_ = .509) age in the unstratified model.

**Table 3:** Estimated adjusted^a^ mean group differences in BDI-II score by age.

	18–39 years		40–59 years		60–82 years	
	Diff (CI)	*d*	Diff (CI)	*d*	Diff (CI)	*d*
**BDI-II**						
Baseline	0.67 (-0.13; 1.48)	–	0.73 (-0.27; 1.73)	–	-0.28 (-2.64; 2.09)	–
6 weeks	-3.08 (-4.91; -1.26)	0.44	-2.26 (-4.22; -0.31)	0.30	-6.53 (-9.57; -3.48)	1.91
6 months	-4.27 (-7.09; -1.46)	0.43	-4.73 (-7.43; -2.04)	0.41	-7.73 (-12.42; -3.05)	1.25
**Interaction**_Group X Time_	F = 8.72, *p* < 0.001		F = 5.96, *p* = 0.003		F = 5.31, *p* = 0.005	
**Interaction**_Group X Time X Age_ ^b^	F = 0.99, *p* = 0.372 (F = 0.83, *p* = 0.509)					

Diff, adjusted mean BDI-II difference, i.e., IG minus CG; CI, 95% confidence interval; d, standardized mean difference (Cohen’s d).

a Age-stratified multilevel mixed-effects linear regression models were used to estimate BDI-II score at follow-up from treatment group (IG vs. CG), time, and interaction between treatment group and time. Analyses adjusted for baseline BDI-II score, age, gender, marital status, education, vocational qualification, employment status, domicile, treatment history and comorbid anxiety disorder, as well as for medication intake due to psychological distress at follow-up. Variance estimates corrected for clustering by practice.

b Treatment-by-time-by-age interaction was tested using Wald tests with continuous (categorical) age in the overall unstratified model.

### Remission and Response Rates

6 months remission and response rates on the BDI-II are shown in **Table 4**. In general, rates were consistently higher for the IG compared to the CG. Moreover, rates were largely comparable between the age groups in the IG, with about one-third of IG participants showing remission after 6 months and about half of IG participants showing response. In the CG, remission and response rates tended to decline with increased age.

**Table 4:** Remission and response rates at 6 months follow-up by age.

	18–39 years		40–59 years		60–82 years	
	*n*	%	*n*	%	*n*	%
**Remission**						
CG	19/61	31.2	27/129	20.9	8/44	18.2
IG	38/93	40.9	34/91	37.4	6/16	37.5
Significance	*p* = .222		*p* = .007		*p* = .118	
**Response**						
CG	21/69	30.4	30/146	20.6	9/54	16.7
IG	48/96	50.0	40/96	41.7	9/18	50.0
Significance	*p* = .012		*p* <.001		*p* = .005	

Remission indicates the decrease in BDI-II score at follow-up below the clinical threshold of 9 points for those scoring >9 at baseline (n=434); Response indicates a 50% decrease in BDI-II score at follow-up compared to baseline (n=479). p for difference between CG and IG, as indicated by Pearson’s chi2 test.

### Uptake and Adherence

Log-in data on IG participants indicated that the uptake of the intervention was similar in all three age groups, with around 70% having accessed the iCBT program at least once during the observation period (*χ²* = 0.18, *p* = .915; **Table 5**). Computer records further showed that the number of completed modules increased in older age groups (*χ²* = 18.99, *p* = .040). While 37% (43/115) participants aged 18 to 39 years accessed but did not complete the first module, it was 25% (24/95) in 40–59-year-olds, and 7% in 60+ year-olds. Conversely, having completed all five modules was reported by 10% (11/115) of the youngest, 20% (19/95) of the middle-aged, and 27% (4/15) of the oldest group. Likewise, participants in the oldest group tended to use iCBT more often longer than 6 weeks, although the difference to younger participants was not significant (*χ²* = 3.34, *p* = .188).

**Table 5:** Uptake and adherence in the treatment group by age (*n* = 320).

	18–39 years		40–59 years		60–82 years	
	*n*	%	*n*	%	*n*	%
**Log-in data**						
Uptake						
Yes	115/166	69.3	95/133	71.4	15/21	71.4
No	51/166	30.7	38/133	28.6	6/21	28.6
Completed modules						
1^st^	43/115	37.4	24/95	25.3	1/15	6.7
2^nd^	42/115	36.5	25/95	26.3	4/15	26.7
3^rd^	14/115	12.2	22/95	23.2	4/15	26.7
4^th^	5/115	4.4	5/95	5.3	2/15	13.3
5^th^	11/115	9.6	19/95	20.0	4/15	26.7
Usage time						
up to 6 weeks	101/115	87.8	78/95	82.1	11/15	73.3
longer than 6 weeks	14/115	12.2	17/95	17.9	4/15	26.7

## Discussion

The use of iCBT as a complementary self-management option for routine treatment of mental disorders among the elderly is still an underrepresented topic in research and practice. The present study adds to the sparse findings on older adults in the literature by examining and comparing the effect of self-guided iCBT in mild to moderately severe depressed primary care patients across the lifespan within a large-scale cluster-randomized controlled trial.

This secondary analysis of the trial found no age differences in the effectiveness of iCBT. In all groups, IG patients experienced a greater reduction in the severity of depression after 6 weeks and 6 months than did CG patients. It can be concluded that the intervention was just as effective in over-60s as it was in under-30s, suggesting iCBT as a valuable universal adjunct to the treatment of depression in primary care. In addition, the current study found a large effect size in the oldest age-group (*d* > 1.0). These results are largely in line with the few available and primarily non-RCT studies on iCBT in individuals with late-life depression. For example, Mewton et al. (38) found that iCBT is just as effective in over 60 year-olds as in younger age groups seeking depression and other psychiatric treatment in primary care. The iCBT intervention in their study with 2,413 Australian participants consisted of six self-management online-modules specific to the four disorders major depression, generalized anxiety disorder, panic disorder or social phobia. Their results indicate significant reductions in psychological distress and disability, regardless of age. Likewise, Hobbs et al. (39) investigated the effect of iCBT over the entire lifespan in a large sample (n=1,288) of patients seeking help for depression in routine care. They found moderate to large effects in reducing depression severity, psychological distress, and impairment in favor of iCBT, and that effect sizes were comparable between groups from 18 to 65+ years of age. Titov et al. (33) found similarly large effect sizes (*d* > 1.0) with regard to the reduction of depressive symptoms amongst individuals aged 60 years and older (n=433) by comparing different types of clinician-guided and self-guided iCBT, including five online-modules on homework assignments and case-enhanced stories. The same applies to O´Moore et al. (40) who have tested the efficacy of an iCBT program for depression in individuals with osteoarthritis of the knee and comorbid depression (n=69). Their iCBT intervention consisted of six online-modules, as well as regular homework assignments and access to supplementary resources. Their results revealed that not only the mental well-being improved, but also the osteoarthritis-related self-efficacy, pain, stiffness, and physical function.

The present study makes important contributions to the literature. While the aforementioned studies used naturalistic pre-post designs without follow-up (38, 39), lacked a concurrent control (33), or were based on small and highly specific samples (40), our study relies on a statistically powered sample of patients, who were randomly assigned to either intervention or control and compared in terms of depression severity in both short- and long-term follow-ups across age. This way, the present study provides the first reliable evidence of a sustained beneficial effect of iCBT over TAU on the severity of depression at different stages of life. In addition, IG patients in previous studies often received a high level of support from clinicians, for example, by prescribing iCBT as part of treatment or scheduling frequent GP contacts. We could show that self-guided iCBT that was recommended but not further monitored by the GP, is equally effective in all age groups. This reinforces growing evidence (33) that elaborate clinical guidance may not be imperative to achieve a clinically significant reduction in severity of late-life depression through iCBT. Finally, the majority of previous studies on integrated iCBT in routine treatment of depression among older adults came from Australia. Taken together, there is now convincing evidence that the sustained benefit of iCBT in treating late-life depression is largely unaffected by cultural or healthcare system-related differences.

There has been increasing discussion about the high comorbidity between late-life depression and cognitive impairment in recent years (8–12), and one could speculate whether effective treatment of depressive symptoms also has an impact on neurodegenerative processes in old age. However, studies on the pathogenesis and conditionality of both diseases are challenging and findings are inconsistent or even contradictory (27). There is some evidence, though, that integrated treatment of depression and dementia in late life helps to improve cognitive functioning (41, 42). Whether specifically tailored E-Mental health interventions for late-life depression may also contribute to slowing cognitive decline in the long term and thus help to improve the quality of life in broader terms, should be the focus of future research.

Interestingly, the present study contrasts the results of Dorow et al. (18) who found that preferences for Internet-based interventions as part of depression treatment tended to decrease with age. Moreover, in a qualitative study aiming to investigate the acceptability of online self-management for depression in individuals with obesity, Löbner et al. (43) found that also healthcare professionals consider younger patients as the preferred target group for iCBT use. There seems to be a discrepancy between the expected and actual benefits of iCBT from the perspectives of older adults and treating healthcare professionals. In addition, barriers to the uptake of iCBT, such as low accessibility or negative attitudes toward Internet interventions may also play a role, preventing GPs from recommending and patients from choosing or maintaining offered treatments (44). Taking into account the increasing use of the Internet especially among older people (45), future studies should look into ways to reduce fears and doubts regarding the use of iCBT on both sides. Possible interventions might include target group-specific information campaigns by expert associations and health care insurances to promote the search for help in older adults and educate general practitioners (3). Gender-specific preferences of the elderly should particularly be taken into account in order to improve the need-based access to psychological treatment (46, 47).

Lastly, older participants in the current study tended to show a better adherence to the iCBT program than younger participants, as has been reported by others as well (38, 39, 48). The group over 60 years of age completed more modules and logged in more often than younger age groups, although in general they seem to prefer iCBT less frequently. One possible explanation might be that elderly people simply have more time during the day to spend time on the iCBT training sessions. For instance, we could see that the majority of IG participants in the oldest age group were already retired. In accordance with Karyotaki et al. (49), this group also tended to be better educated, which could have rendered them more compliant and adherent to the iCBT program.

### Limitations

This study is not without limitations. Compared to the groups of younger participants, the older age group (60+) was proportionately smaller in our trial. Moreover, given that an age-stratification of study participants was not intended with regard to randomization procedures within the @ktiv-trial, we also observed an uneven distribution of iCBT across age strata, with fewer IG allocations in the oldest group. Nonetheless, iCBT uptake and usage behavior were comparable between the age groups and even point to a more intensive use of iCBT among the small group of 60+ year-olds. Future studies are needed that take randomized controlled trial designs with specific age-stratifications into account. We further agree with previous conclusions (24) that future iCBT trials should involve even older age groups (80+) and that age should increasingly become the focus as a moderator of treatment effects in iCBT research. In addition, data on medication intake as part of TAU during follow-up were self-reported which could limit its reliability. Finally, the @ktiv trial excluded individuals with no Internet skills and no access to the Internet, which further limits the generalizability of results. In fact, future studies should comprise more people with low computer or Internet literacy in order to find out whether this group might also benefit from iCBT interventions and what guidance is needed to give these individuals proper support.

### Implications

Does Internet-based cognitive behavior therapy suit only for the young? The present study clearly indicates that elderly benefit as much or even more from iCBT as an adjunct element of depression treatment in primary care as do the young. Older adults increasingly use the Internet and therefore should not be left out when discussing iCBT as treatment option. Possible fears and doubts with regard to the use of iCBT in both elderly depressed individuals and their health care professionals should be tackled by suitable interventions, such as information campaigns. Further research is needed to elaborate implementation strategies that work best for the elderly.

## Data Availability Statement 

The datasets for this manuscript are not publicly available due to patient confidentiality and participant privacy. Requests to access the datasets should be directed to the corresponding author and are available upon reasonable request.

## Ethics Statement

The studies involving human participants were reviewed and approved by IRBs of the University of Leipzig (reference number 222/14ff) and of the Australian National University (reference number 2013/342). The patients/participants provided their written informed consent to participate in this study.

## Author Contributions

AP formulated the research question, wrote the statistical analysis plan, conducted all statistical analyses, interpreted the data, wrote the manuscript, and gave final approval of the version to be published. MLö substantially contributed to the acquisition of the data, supported in interpreting the data and drafting the manuscript, and gave final approval of the version to be published. JS, MLu, AK, and H-HK substantially contributed to acquisition of the data, revised the manuscript critically for important intellectual content, and gave final approval of the version to be published. SR-H conceptualized and designed the study, supported in interpreting the data, revised the manuscript critically for important intellectual content, and gave final approval of the version to be published.

## Funding 

This work was supported by the Federal Association of AOK (Project number BGAAF-0608); and is part of the study “Improving care of late-life depression: acceptability and effectiveness of the web-based self-management program trauer@ktiv” that was funded by the German Federal Ministry of Education and Research (AgE-health, grant number: 01GY1613). The funder of the study contributed to the trial design, but had no role in patient recruitment and in conducting the trial including collection, analysis and interpretation of the data, or writing and revising the manuscript.

## Conflict of Interest

The authors declare that the research was conducted in the absence of any commercial or financial relationships that could be construed as a potential conflict of interest.
